# Combination of Innate Immune Modulators as Vaccine Adjuvants in Mice

**DOI:** 10.3390/vaccines8040569

**Published:** 2020-10-01

**Authors:** Azita Haddadi, Alyssa Chaffey, Siew Hon Ng, Damayanthi Yalamati, Heather L. Wilson

**Affiliations:** 1 Division of Pharmacy, College of Pharmacy & Nutrition, University of Saskatchewan, Saskatoon, SK S7N 5E5, Canada; Azita.Haddadi@usask.ca; 2 Vaccine and Infectious Disease Organization-International Vaccine Centre (VIDO-InterVac), University of Saskatchewan, Saskatoon, SK S7N 5E3, Canada; alyssa.chaffey@usask.ca (A.C.); siewhon.ng@usask.ca (S.H.N.); 3 Alberta Research Chemicals Inc., Edmonton, AB T6G 2M9, Canada; dyalamati4@arciglobal.com

**Keywords:** mice, adjuvant, muramyl dipeptide, PAM3CSK4, MPLA analogue, PLGA nanoparticles

## Abstract

The development of new, effective, and safe vaccines necessarily requires the identification of new adjuvant(s) to enhance the potency and longevity of antigen-specific immune responses. In the present study, we compare the antibody-mediated and cell-mediated immune (CMI) responses within groups of mice vaccinated subcutaneously with ovalbumin (OVA; as an experimental antigen) plus polyphosphazene (an innate immune modulator), Polyinosinic:polycytidylic acid (poly-I:C; (an RNA mimetic) and glycopeptide ARC5 (which is a Toll-like receptor (TLR), TLR2 ligand and PAM3CSK4 analogue) formulated together in a soluble vaccine. We also investigated the effect of a polymeric nanoparticle of ARC4 and ARC7 (which are a novel muramyl dipeptide analogue and a monophosophoryl lipid A (MPLA) analogue, respectively) plus OVA +/− ARC5 as a subcutaneous vaccine in mice. OVA+ARC4/ARC7 nanoparticle +/− ARC5 triggered a robust and balanced Th1/Th2-type humoral response with significant anti-OVA IgA in serum, and significant interferon (IFN)-γ and interleukin (IL)-17 production in splenocytes after 35 days relative to the controls. Formulation of OVA with ARC4/ARC7 nanoparticles should be investigated for inducing protective immunity against infectious pathogens in mice and other species.

## 1. Introduction

Vaccines continue to be an important public health tool in the control of infectious diseases and they are estimated to prevent approximately 2.5 million deaths and many more illness each year worldwide [1]. A major trend in vaccinology has been to use subunit vaccines, as they cannot revert back to a pathogenic form and are therefore a safe vaccine choice, especially for those who are immunocompromised. However, because subunit antigens are highly purified, they tend to be poorly immunogenic and they must be formulated with adjuvants to induce strong immunity [2]. Most commercial vaccines are formulated with a single adjuvant but combination adjuvants can fine-tune and selectively direct the type or augment the magnitude of the immune response. Combinational adjuvants may be particularly beneficial for vaccines against specific populations, such as newborns, who generally have a default Th2 response, and the elderly, who tend to have decreased immune responses partly as a result of thymic involution with advancing age [3]. Rigorous safety evaluations must be performed on each new vaccine formulation, as strong adjuvants have the potential to induce undesirable side effects such as inflammation at the injection site [4,5].

Our research group investigated the effect that polyphosphazene (PZ), polyinosinic:polycytidylic acid (PIC), and the innate immune modulators ARC4, ARC5, and ARC7 have on the immune response. PZs are a class of synthetic polymers consisting of a backbone of alternating phosphorus and nitrogen atoms and organic side groups attached to each phosphorus atom. PZ has been shown to affect the gene expression of NLRP3, the inflammasome receptor [6], the production of pro-inflammatory cytokines interleukin (IL)-1β and IL-18 at the site of injection [6,7], and DC maturation and activation [8,9,10]. PIC is a synthetic double-stranded RNA and Toll-like receptor (TLR), TLR3 ligand with adjuvant potential [11]. ARC4 is a glycolipopeptide that is a muramyl dipeptide (MDP) analogue and a NOD2 agonist. MDP was first identified in the bacterial cell wall component peptidoglycan. In cells, MDP is bound by NOD2, a cytoplasmic receptor belonging to the innate immune system [12]. MDP induces immune responses by increasing the production of interferon (IFN)-γ and other cytokines [13], stimulating the differentiation and proliferation of lymphocytes [14] and it has been shown to influence immune responses with other TLR ligands [15]. ARC5 is a TLR2 ligand and PAM3CSK4 analogue. PAM3CSK4 has been shown to activate TLR1/TLR2 heterodimers and induce cytokine production [16] and lymphocyte activation [17]. Here, we investigate whether TLR agonists and immunomodulators alone or as part of a poly-(lactic-co-glycolic acid) (PLGA) nanoparticle (NP) delivery vehicle can act as effective vaccine adjuvants. PLGA NPs have shown promise as potential delivery vehicles for immune modulators [18,19,20,21,22], proteins, and peptides [18,19,22]. ARC7 is a glycolipid that is also a TLR4 ligand and a monophosphoryl lipid A (MPLA) analogue. MPLA is a detoxified derivative of lipopolysaccharide (LPS) that has an immunomodulatory impact on the innate and adaptive immune system, and has been used as a vaccine adjuvant in humans [23]. Here, we evaluated whether multiple adjuvants can augment humoral immunity or cell-mediated immunity (CMI) to ovalbumin in mice. A new and highly effective vaccine adjuvant combination could be beneficial for the development of new vaccines for veterinary species and human diseases.

## 2. Materials and Methods

### 2.1. Ethics Statement 

All animal experiments were conducted according to the Guidelines for the Care and Use of Laboratory Animals as indicated by the Canadian Council on Animal Care and were approved by the Animal Care Committee of the University of Saskatchewan. 

### 2.2. Vaccine Components 

ARC4 is a glycolipopeptide that is a NOD2 agonist and an MDP analogue. ARC5 is a lipopeptide that is a TLR2 ligand and a PAM3CSK4 analogue. ARC7 is a glycolipid that is also a TLR4 ligand and an MPLA analogue. ARC4, ARC5, and ARC7 were synthesized and purified by Alberta Research Chemicals Inc. (Edmonton, AB, Canada) through proprietary means. Poly-(di(sodium carboxylatoethylphenoxy)-phosphazene) (PZ) was purchased from Idaho National Laboratory (Idaho Falls, ID, USA). Poly I:C was purchased from Invivogen (San Diego, CA, USA). HDP was purchased from Genscript (Piscataway Township, NJ, USA) at 90% purity. Ovalbumin was purchased from Sigma-Aldrich. The vaccine diluent was PBS, pH 7.4 (Gibco, Life Technologies, Gaithersburg, MD, USA).

### 2.3. Nanoparticle Formulation 

PLGA NPs were prepared by the emulsification solvent evaporation method as mentioned previously [24]. Briefly, an OVA/PBS solution (10%), ARC7 in chloroform:methanol (2%), or ARC4 in chloroform:methanol (2%) was transferred to the PLGA/chloroform solution (25%). The resulting mixtures were then emulsified in 5% of polyvinyl alcohol (PVA) to form a secondary emulsion followed by stirring for 2 h to evaporate the solvents. The NPs were then collected by centrifugation. At the final step, the NPs were freeze-dried and stored at −20 °C for further use. In preliminary studies, various amounts of NPs containing adjuvants were mixed at different ratios to obtain the required dose of vaccine for immunization. The amounts were calculated based on the loading efficiency of the antigen and adjuvants in every batch of NPs.

### 2.4. Evaluation of the Loading Efficiency

The amounts of adjuvants encapsulated in the PLGA NPs were determined by LC-MS/MS using a pre-column guard (Eclipse XDB-Rapid resolution, C8, 2.1 Å~ 30 mm, 3.5 μm; Agilent, Santa Clara, CA, USA). For ARC7 encapsulation, a previously published method from our group was applied [25]. To analyze ARC4 encapsulation, a high-performance liquid chromatography (HPLC) system was interfaced with the AB Sciex 4000 hybrid triple quadrupole linear ion trap mass spectrometer. Applied Biosystems/MDS Sciex Analyst software (Version 1.6.0, Framinham, USA) was used for system control and quantification. A sample volume of 5 µL was injected using the 1200 Agilent auto injector set to 4 °C and was delivered with an isocratic mobile phase consisting of methanol (0.1% formic acid) at a flow rate of 200 µL/min for a run-time of 2 min. A BCA assay was used to quantify the amount of OVA in NP formulations, according to our previous studies [18].

### 2.5. Mouse Immunization

Six- to eight-week-old BALB/C mice were obtained and randomly assigned to groups one week prior to experimentation to allow them to acclimatize. Mice (*n* = 8 per group) were immunized subcutaneously with 200 μL total volume of OVA alone, OVA+ARC5, OVA+PZ+PIC, OVA+ARC5+PZ+PI, OVA+ARC4-ARC7 nanoparticles, or OVA+ARC5+ARC4-ARC7 nanoparticles. The concentration of OVA was 50 μg/dose and the adjuvants were as follows: PZ (10 μg/dose), PIC (10 μg/dose), ARC5 (20 μg/dose), ARC7 (16 μg/dose), and ARC4 (3.1 μg/dose). Mice received a booster immunization on Day 21. Blood was obtained on Day 21 and Day 35 and spleens were excised when the mice were euthanized. Mice were assessed for immune site reactions daily and none was evident in any of the groups of mice throughout the trial. 

### 2.6. Splenocyte Isolation and Ex Vivo Restimulation

Spleens were excised and incubated in minimal essential media (MEM) (Sigma Life Science). They were placed on a petri dish, minced into smaller pieces, and pushed through a 40 μm nylon cell strainer (Corning Life Sciences). The medium was used to rinse the splenocytes from the strainer as well as the petri dishes. Splenocytes were centrifuged at 350× *g* for 10 min at 10 °C. The supernatant was discarded and cells were incubated in Gey’s solution (CaCl_2_ (0.220 g), KCl (0.370 g), KH_2_PO_4_ (0.03 g), MgCl_2_ (0.210 g), MgSO_4_ (0.070 g), NaCl (8.000 g), NaHCO_3_ (0.227 g), Na_2_HPO_4_ (0.120 g), D-glucose (1.000 g) in 1 L distilled water) for 10 min at room temperature (RT) to lyse any red blood cells. Cell were centrifuged at 350× *g* for 10 min at 10 °C, and the supernatants were discarded and replaced with AIM V (Gibco, Life Technologies) plus 10% fetal bovine serum (FBS; Gibco, Life Technologies). Cells were counted manually using a hemocytometer and Trypan Blue reagent (Gibco, Life Technologies) using standard techniques. Splenocytes were diluted with prewarmed AIM V (Gibco) and 10% FBS (Gibco) to a density of 1.0 × 10^6^ cells per well (ELISA) into 96-well tissue culture plates (Thermofisher) and placed in a 37 °C 5% CO_2_ incubator for 1 hour to stabilize before stimulation with OVA or media (vaccine trial) or stimulation with adjuvants to measure the effect of the immunostimulants on primary splenocytes. 

Cells were mock-stimulated with media or stimulated with OVA (1 μg/mL) for 48 h then supernatants were frozen at −20 °C until they were subjected to IL-4, IL-10, IL-17, or IFN-γ cytokine ELISAs. 

### 2.7. Lymphocyte Proliferative Response Assay

Cells were cultured in 96-well flat-bottom plates (Nalge Nunc International, Naperville, IL, USA) at 1 × 10^5^ cells/well and 10% CD14^+^ myeloid cells (as indicated) in a final volume of 200 μL culture medium with triplicate wells. Cells were incubated for 72 h followed by the addition of 0.4 μCi ^3^H-thymidine (Amersham Pharmacia Biotech)/well for another 16 h of culture. Cells were freeze–thawed and harvested onto Unifilter plates (Perkin–Elmer, Boston, MA, USA) and incorporation of ^3^H-thymidine was measured as counts per minute (cpm) using a liquid scintillation counter (Top-Count, Perkin–Elmer). Each experiment was performed separately with cells isolated from several animals as indicated. 

### 2.8. Cytokine Detection by ELISA in Mice

For ELISA analysis, R&D Systems Duoset kits (Fisher Scientific) were used to quantify IL-4, IL-10, IL-17, and IFN-γ as per the manufacturer’s protocol. All assays were performed on Immulon 2, 96-well microtiter plates (Dynex Technology Inc., Chantilly, VA, USA). Limit of detection for each cytokine in each experiment is indicated in the Figure legend. 

### 2.9. Statistical Analysis

Statistical analyses were carried out using Graph-Pad Prism 6 software (GraphPad Software, San Diego, CA, USA). Differences in the cytokine production were identified using a non-parametric Kruskal–Wallis ANOVA test where Dunn’s multiple comparisons test was used post-hoc to identify statistically significant differences in cytokine production. Differences in the frequency of OVA-stimulated or unstimulated CD4^+^ T cells were determined using Wilcoxon *t*-tests and differences across treatments were determined using Kruskal–Wallis ANOVA tests as above. Differences were considered statistically significant at *p* < 0.05, *p* < 0.01, *p* < 0.001, and *p* < 0.0001 as stated in the text.

## 3. Results

**Figure 1:**
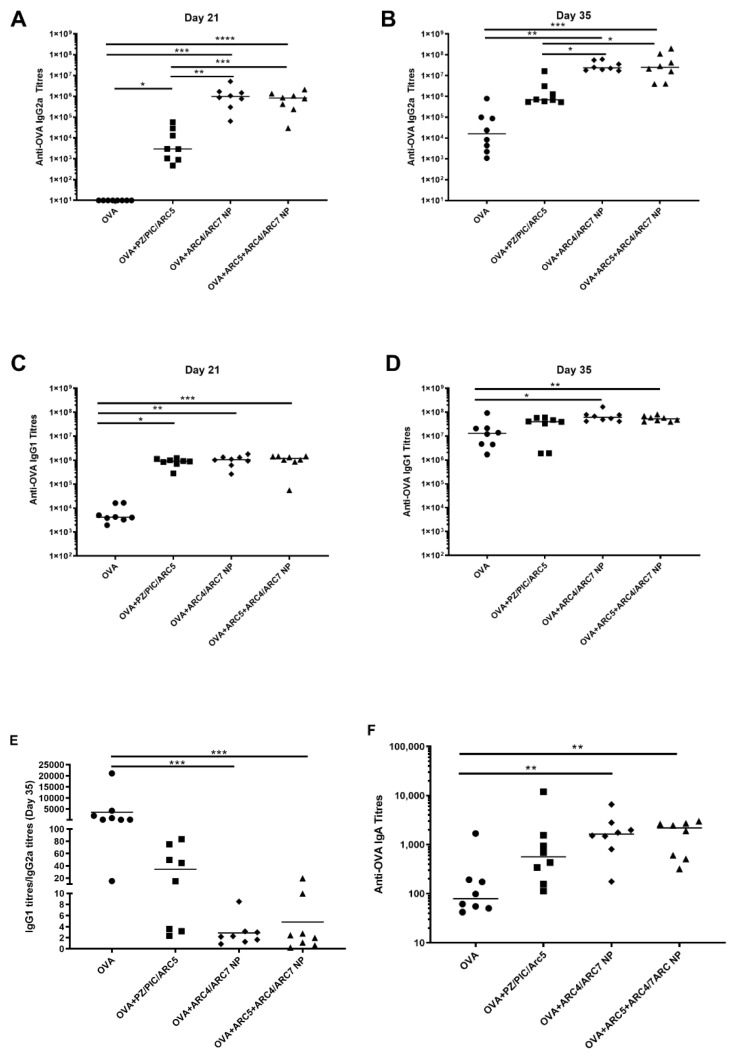
OVA-specific humoral immune responses in mice immunized with OVA co-formulated with ARC5, polyphosphazene (PZ), Poly I:C, or ARC4/ARC7 nanoparticles. BALB/C mice (*n* = 8) were immunized on Day 1 and Day 21 with soluble OVA (50 µg/dose) alone or formulated with PZ (10 µg/dose) + Poly I:C (10 µg/dose) + ARC5 (10 µg/dose) (PZ/PIC/ARC5), OVA+ARC5+1:1 ARC4/ARC7 or OVA+1:1 ARC4/ARC7. OVA-specific serum IgG2a titers were measured on Day 21 (**A**) and Day 35 (**B**) and OVA-specific IgG1 titers were measure on Day 21 (**C**) and Day 35 (**D**). Ratio of IgG2a/IgG titres on day 35 are shown in (**E**). OVA-specific serum IgA titers were measured on Day 35 (**F**). ELISA titers are expressed as the reciprocal of the highest dilution resulting in a reading of two standard deviations above the negative control. Each data point represents an individual animal and median values are indicated by horizontal lines. Statistical analysis was performed by one-way ANOVA and the differences between the treatments were compared by Kruskal–Wallis tests and Dunn’s multiple comparison test. Significant differences are depicted as follows: * *p* < 0.05, ** *p* < 0.01, *** *p* < 0.001, **** *p* < 0.0001.

Mice were immunized through the subcutaneous route with OVA alone, the OVA+PZ/PIC/ARC5 group, or OVA+ARC4/ARC7 NPs with or without ARC5. The results from mass spectrometry showed that the loadings for ARC7 and ARC4 were 1.79 and 0.30 μg/mg of NP, respectively. According to a BCA performed to quantify OVA in the NPs, the loading efficiency was found to be 7.5 μg/mg of NPs. The average particle size for all the NPs in this study was found to be about 200 nm and the surface charge was slightly negative to neutral (data not shown). All responses are relative to mice immunized with OVA alone. Mice immunized with OVA+ARC4/ARC7 NP (Figure 1A; *p* < 0.0001, *p* < 0.001) and OVA+ARC5+ARC4/ARC7 NP (Figure 1A; *p* < 0.001, *p* < 0.01) showed significantly higher anti-OVA IgG2a titers after the first immunization (Day 21) and after the booster vaccine (Day 35), respectively. Both NP formulations showed significantly higher anti-OVA IgG2a relative to the mice immunized with OVA+PZ/PIC/ARC5 on Day 21 (Figure 1A; *p* < 0.01, *p* < 0.001) and Day 35 (Figure 1B; *p* < 0.05, *p* < 0.05), respectively. Mice immunized with OVA+ARC4/ARC7 NP (Figure 1C; *p* < 0.01, *p* < 0.05) and OVA+ARC5+ARC4/ARC7 NP (Figure 1D; *p* < 0.001, *p* < 0.01) had significantly higher anti-OVA IgG1 titers after Days 21 and 35, respectively. Mice immunized with OVA+PZ/PIC/ARC5 (*p* < 0.05) had significantly higher anti-OVA IgG1 titers after the first immunization (Day 21; Figure 1C) relative to mice immunized with OVA but this significant difference was not conserved after the booster immunization (Figure 1D). Neither NP formulation resulted in significantly higher anti-OVA IgG1 titers relative to the mice immunized with OVA+PZ/PIC/ARC5. A balanced Th1/Th2-type humoral immune response would have a ratio of IgG1:IgG2a titers of 1. When IgG1 titers were divided by the IgG2a titers, the ratios were as follows (Figure 1E): mice immunized with OVA showed 3596.4 (±7211.5) titers, mice immunized with OVA+PZ/PIC/ARC5 showed 34.7 (±33.3) titers, mice immunized with OVA+ARC5+ARC4/ARC7 NP showed 2.8 (±2.4) titers and mice immunized with OVA+ARC4/ARC7 NP showed 4.9 (±6.8) titers. The NP formulations induced a more balanced Th1/Th2-type humoral response. Finally, serum anti-OVA IgA titers were significantly higher in mice immunized with either NP formulation relative to OVA (Figure 1F; *p* < 0.01, *p* < 0.01). Further analyses will need to be performed on mucosal tissues to determine whether either NP formulation increased mucosal IgA antibody titers. Collectively, these data indicate that the NP formulations triggered a balanced Th1/Th2 serum antibody response and IgA serum response. The addition of ARC5 to the ARC4/ARC7-NP did not significantly impact the antibody-mediated immune response. 

**Figure 2:**
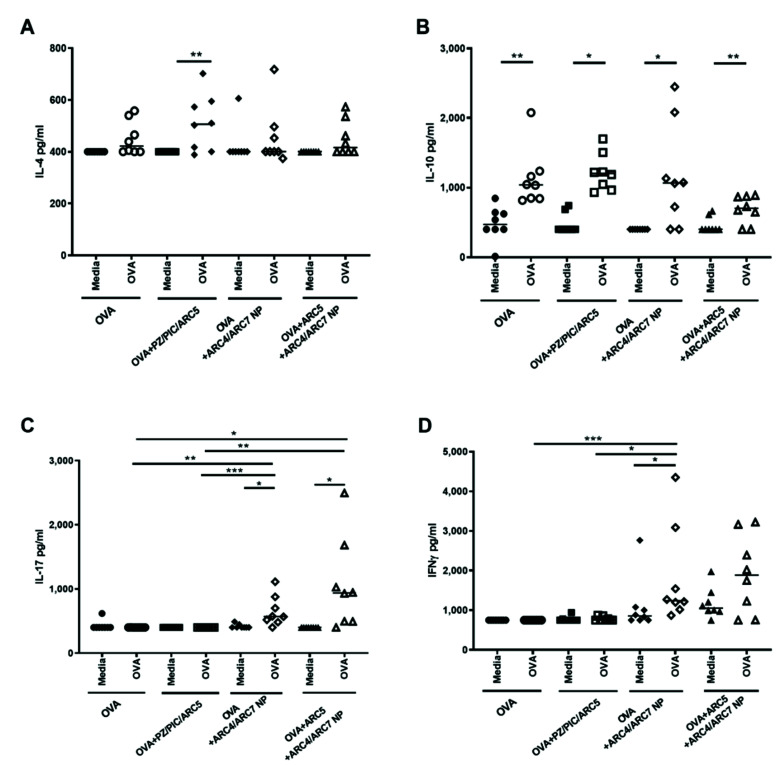

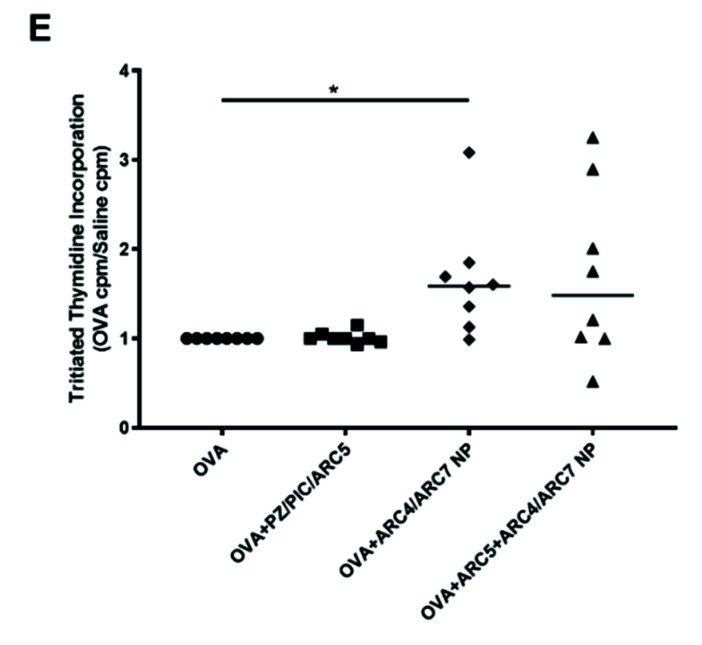
OVA-specific Th1- or Th2-biased immune responses in mice immunized with OVA co-formulated with ARC5, PZ, Poly I:C (PIC), or ARC4/ARC7 nanoparticles. BALB/C mice (*n* = 8) were immunized on Days 1 and 21 with soluble OVA (50 µg/dose) alone or formulated with PZ (10 µg/dose) + Poly I:C (10 µg/dose) + ARC5 (10 µg/dose) (PZ/PIC/ARC5), OVA+ARC5+1:1 ARC4/ARC7 or OVA+1:1 ARC4/ARC7. IL-4 (**A**), IL-10 (**B**), IL-17 (**C**), and IFN-γ (**D**). Cytokine production by splenocytes restimulated in vitro with OVA or media was measured using ELISA analysis on Day 48. (**E**) Lymphocyte proliferation was measured by culturing splenocytes restimulated with OVA or media for 36 h, then tritiated thymdine was added to the media for another 18 h. Radioactive thymidine incorporation into cells indicates B and T lymphocyte proliferation. Each data point represents OVA-stimulated cells/media-stimulated cells from each individual animal. Median values are indicated by horizontal lines. Statistical analysis was performed by one-way ANOVA and the differences between the treatments were compared by Kruskal–Wallis tests and Dunn’s multiple comparison test. Statistical differences between media and OVA-restimulated splenocytes were calculated using Student’s *t*-test.: * *p* < 0.05, ** *p* < 0.01, *** *p* < 0.001.

To assess the impact of the vaccines on the murine cell-mediated immune response, we measured the OVA-specific secretion of IL-4 (Figure 2A), IL-10 (Figure 2B), IL-17 (Figure 2C), and IFN-γ. Cells from the mice immunized with OVA+PZ/PIC/ARC5 that were restimulated with OVA had significantly higher IL-4 (*p* < 0.01) and IL-10 (*p* < 0.05) relative to mock-immunized cells from each group of mice. Significant OVA-specific IL-10 (*p* < 0.05), IL-17 (*p* < 0.05) and IFN-γ (*p* < 0.05) were induced in mice vaccinated with OVA+ARC4/ARC7 NP. Significant OVA-specific IL-10 (*p* < 0.01) and IL-17 (0.05), but not IFN-γ, were induced in mice vaccinated with OVA+ARC5+ARC4/ARC7 NP. Importantly, neither NP formulation triggered significantly higher IL-10 relative to the OVA-immunized mice. OVA-stimulated cells from mice immunized with OVA+ARC5+ARC4/ARC7 NP produced significantly higher IL-17 relative to OVA-stimulated cells from mice immunized with OVA alone (*p* < 0.05) or with OVA+PZ/PIC/ARC5 (*p* < 0.01). Likewise, mice immunized with OVA+ARC/ARC7 NP had significantly higher secretion of IL-17 relative to OVA-stimulated cells from mice immunized with OVA alone (*p* < 0.01) or with OVA+PZ/PIC/ARC5 (*p* < 0.001). Finally, mice immunized with OVA+ARC4/ARC7 NP had significantly higher OVA-stimulated IFN-γ production relative to mice immunized with OVA alone (*p* < 0.001) or OVA+PZ/PIC/ARC5 (*p* < 0.5). Only the mice immunized with OVA+ARC4/ARC7 NP showed significantly higher lymphocyte proliferation relative to OVA-immunized mice (Figure 2E; *p* < 0.05).

## 4. Discussion

PLGA NPs act as potential delivery systems for several classes of drugs and therapeutic agents. We have previously shown the pronounced effect of PLGA nanoparticles in delivering anticancer agents [18,26,27], immune modulators [18,19,20,21,22], small molecules [28], proteins, and peptides [18,19,22] to different cells and organs in vitro and in vivo. PLGA NPs have shown to be promising carriers, particularly for future cancer vaccine formulations [18,19,20,21,24,29]. Modification of the physical properties of PLGA NPs could shift the delivery of encapsulated antigens to either the cytoplasm (for MHC I presentation and CD8^+^ T-cell activation) or to the endosome (for MHC II presentation and CD4^+^ T-cell activation) [22]. According to previous studies, cytoplasmic delivery of PLGA content is affected by differences in the molecular weight of PLGA. Investigations into modifying PLGA formulations for optimum cellular and humoral immunity will assist researchers to design immunization strategies capable of activating robust CD4^+^, CD8^+^ T-cells, Nature Killer (NK) cells, and NK T cells that could mediate full-scale antitumor immune responses [30]. 

Previously, we showed that mice immunized with OVA formulated with single adjuvants such as CpG, PZ, or an host defense peptide (HDP) failed to trigger significant anti-OVA IgG serum titers of IFN-γ or IL-5 cytokine expression [4]. In the current experiments, BALB/C mice immunized with OVA+PZ/PIC/ARC5 showed significant and comparable OVA-specific IgG2a and IgG1 over time. However, these vaccines failed to promote strong induction of OVA-specific IL-4, IL-10, IL-17, and IFN-γ, which is somewhat inconsistent with previous results in mice. Specifically, we previously showed that C57Bl/6 mice immunized with 10 µg OVA plus a triple adjuvant formulation with 10 µg CpG/20 µg HDP/10 µg PZ induced significant anti-OVA IgG production and IFN-γ secretion [4,31]. Whether the presence of CpG dinucleotide (CpG) instead of PIC or our five-fold increased dose of soluble OVA (50 µg) impacted the response away from triggering OVA-specific IFN-γ secretion is unclear. More likely, the use of BALB/C mice instead of C57Bl/6 impacted the overall immune response but even then, the BALB/C mice are more predisposed to respond with a Th1-type immune response, whereas C57Bl/6 mice are more predisposed to respond to vaccines with Th2-type immunity [32]. We observed that a modest but statistically significant induction of OVA-specific IL-4 and IL-10 production was induced in BALB/C mice immunized with OVA+PZ/PIC/ARC5. The humoral immune response to OVA+PZ/PIC/ARC5 succeeded in inducing anti-OVA IgG2a titers (a Th1-type immune response) and IgG1 titers (a Th2-type immune response). Overall, the soluble vaccine formulated with PZ, PIC, and ARC5 glycopeptide provided a Th1/Th2-type immune response with limited but significant IL-4 or IL-10 cytokine production in BALB/C mice.

Formulation of the vaccine to include PLGA NPs of ARC4/ARC7 +/− ARC5 led to significantly higher anti-OVA IgG2a titers relative to mice immunized with either OVA or OVA+PZ/PIC/ARC5. When we compared the OVA-specific IgG1 and IgG2a titers, the NP formulations produced ratios of approximately five and two, respectively, which is a very balanced Th1/Th2-type immune response. This level of balanced Th1/Th2-type humoral immunity is potentially interesting for neonatal vaccines, wherein the immune response is naturally skewed toward Th2-type immunity. Both NP formulations also showed superior induction of serum anti-OVA IgA, and more experiments should be performed to determine whether the mucosal immune response is similarly elevated. Inclusion of ARC5 failed to promote a significant induction of OVA-specific antibodies relative to the OVA+ARC4/ARC7 vaccines, meaning that ARC5 may not be a necessary adjuvant to promote humoral immunity against this antigen in BALB/C mice. Lymphocyte proliferation indicates a cell-mediated immune response if the T cells are proliferating or a humoral response if the B cells are proliferating. Splenocytes from mice vaccinated with OVA+ARC5/PIC/PZ or either of the NPs did not trigger significantly induced OVA-specific LPR relative to the OVA-vaccinated mice, but the vaccine OVA+ARC4-ARC7 induced significant lymphocyte proliferation relative to the unadjuvanted vaccine. 

Importantly, splenocytes from mice vaccinated with OVA+ARC4/ARC7 +/− ARC5 showed significant induction of IL-17, which indicated that ARC4-ARC7 NPs may be inducers of Th17-type immunity, which may be beneficial for antifungal vaccines. Splenocytes from mice immunized with OVA+ARC4/ARC7 NPs showed significant IFN-γ production in response to OVA, which indicates induction of a Th1-type cell-mediated immune response. In contrast, splenocytes from mice vaccinated with OVA+ARC5+ARC4/ARC7 NPs did not respond to OVA restimulation, with significantly higher IFN-γ relative to the cells mock-stimulated with media. These results indicated that PLGA NPs of ARC4/ARC7 may be inducers of a mixed Th1/Th17-type cell-mediated immune response. 

## 5. Conclusions

Collectively, these results indicate that vaccination with OVA+PZ/PIC/ARC5 or ARC4/ARC7 (+/− ARC5) induced significant humoral immunity in mice serum over time but only mice vaccinated with ARC4/ARC7 (+/− ARC5) showed a significant induction of Th1/Th17-type cell-mediated immunity and proliferative lymphocyte response. We conclude that the ARC4/ARC7 NPs are a superior adjuvant combination for inducing humoral and cell-mediated immunity.
